# Risk-Aware Distributionally Robust Optimization for Mobile Edge Computation Task Offloading in the Space–Air–Ground Integrated Network

**DOI:** 10.3390/s23125729

**Published:** 2023-06-20

**Authors:** Zhiyuan Li, Pinrun Chen

**Affiliations:** 1 School of Computer Science and Telecommunication Engineering, Jiangsu University, Zhenjiang 212013, China; 2212108020@stmail.ujs.edu.cn; 2 Jiangsu Provincial Key Laboratory of Industrial Network Security Technology, Zhenjiang 212013, China; 3 Jiangsu Ubiquitous Data Intelligent Perception and Analysis Application Engineering Research Center, Zhenjiang 212013, China

**Keywords:** space–air–ground integrated network, mobile edge task offloading, distributionally robust optimization, conditional value at risk

## Abstract

As an emerging network paradigm, the space–air–ground integrated network (SAGIN) has garnered attention from academia and industry. That is because SAGIN can implement seamless global coverage and connections among electronic devices in space, air, and ground spaces. Additionally, the shortage of computing and storage resources in mobile devices greatly impacts the quality of experiences for intelligent applications. Hence, we plan to integrate SAGIN as an abundant resource pool into mobile edge computing environments (MECs). To facilitate efficient processing, we need to solve the optimal task offloading decisions. In contrast to existing MEC task offloading solutions, we have to face some new challenges, such as the fluctuation of processing capabilities for edge computing nodes, the uncertainty of transmission latency caused by heterogeneous network protocols, the uncertain amount of uploaded tasks during a period, and so on. In this paper, we first describe the task offloading decision problem in environments characterized by these new challenges. However, we cannot use standard robust optimization and stochastic optimization methods to obtain optimal results under uncertain network environments. In this paper, we propose the ‘condition value at risk-aware distributionally robust optimization’ algorithm for task offloading, denoted as RADROO, to solve the task offloading decision problem. RADROO combines the distributionally robust optimization and the condition value at risk model to achieve optimal results. We evaluated our approach in simulated SAGIN environments, considering confidence intervals, the number of mobile task offloading instances, and various parameters. We compare our proposed RADROO algorithm with state-of-the-art algorithms, such as the standard robust optimization algorithm, the stochastic optimization algorithm, the DRO algorithm, and the Brute algorithm. The experimental results show that RADROO can achieve a sub-optimal mobile task offloading decision. Overall, RADROO is more robust than others to the new challenges mentioned above in SAGIN.

## 1. Introduction

With the booming development of the Internet of Things (IoT) ecosystem, users have higher expectations for quality experiences (QoE) across various IoT applications. Following the official deployment and operation of the fifth-generation (5G) mobile system, the sixth-generation (6G) mobile system has gradually come into the limelight. High-speed communication technologies can provide a higher quality of service (QoS) for IoT applications, such as intelligent transportation, intelligent agriculture, maritime surveillance, smart cities, natural disaster relief, and so on. However, existing terrestrial communication networks cannot effectively provide QoS and QoE guarantees for intelligent and low-latency IoT applications.

On the other hand, computing capacity and energy resources in edge nodes are often insufficient to meet the demands of all edge tasks [1]. Task offloading decisions are largely influenced by these factors. Some researchers have addressed these issues from the perspective of an edge–cloud collaboration [2]. Researchers have also turned their attention to the space–air–ground integrated network (SAGIN) to allocate more resources. SAGIN is an integration of the space layer, aerial layer, and ground layer. As a multidimensional network, SAGIN adopts different communication protocols in each segment or integrates different segments to achieve high throughput and reliable data delivery [3].

However, we have to face some new challenges in SAGIN, such as computationally intensive task offloading at the mobile edge and resource allocation in uncertain and heterogeneous network environments. The uncertain network parameters [4], such as uncertain latency, the uncertain amount of arrival tasks [5], and uncertain computation resources, may seriously impact the efficiency of edge task execution. The heterogeneous environment necessitates considering additional factors, such as selecting suitable frequency bands for task propagation and choosing different types of computing nodes. At the same time, the fluctuating processing capabilities make it challenging to allocate appropriate computing resources based on uncertain data when there is a surge of computational tasks. Nowadays, stochastic optimization (SO) [6] and robust optimization (RO) [7,8,9] methods are proposed to solve uncertainty problems. The SO method can use the probability distribution of measuring parameters to predict the potential uncertainty and obtain the mathematical expectation of the objective function. The RO method can directly solve the target value under the worst-case conditions, and it does not need to obtain the probability distributions of measuring parameters. Nonetheless, the results obtained from the SO algorithm may not accurately reflect realistic worst-case scenarios, while the RO algorithm sacrifices a significant amount of performance to obtain computation offloading decisions for worst-case situations. Distributionally robust optimization (DRO) can be viewed as a unifying framework for the SO and RO methods.

The DRO method can replace the probability distribution of uncertain measuring parameters with a fuzzy set. [10]. Then it can choose the worst case in the fuzzy set to obtain better robustness. Additionally, the authors of [11] proposed the conditional value at risk (CVaR) aware DRO method, which can reflect the potential risks and improve stability. In SAGIN environments, the number of tasks requiring offloading may vary, and the availability of computational resources can fluctuate in real time. These uncertainties pose challenges in making efficient task offloading decisions. The risk-aware distributionally robust optimization task offloading (RaDROO) algorithm addresses these challenges by incorporating robust optimization techniques and considering the uncertainties in both the number of tasks and real-time computational resource availability. The two-stage offloading decision process of the RaDROO algorithm enables it to dynamically determine the most suitable task offloading targets. This process considers various factors, such as task characteristics, resource availability, performance requirements, and uncertainties associated with the number of tasks and computational resource availability.

There are two main technical challenges in solving the risk-aware two-stage DRO problem. Firstly, it is difficult to solve the DRO problem due to the fuzzy sets. Secondly, the first-stage offloading decision involves zero-one integer programming. The second stage involves a resource allocation procedure for latency-insensitive and computation-intensive tasks. However, in this paper, we have to solve the risk-aware two-stage DRO problem, which brings new challenges. If we can solve the risk-aware two-stage DRO problem, we can achieve improved revenue while considering uncertainties and ensuring QoS in the SAGIN network architecture.

Our contributions are summarized as follows:

• We investigate the task offloading decision model in the SAGIN environment. The task offloading model consists of two stages: the first stage involves the task offloading decision, while the second stage focuses on edge–cloud collaboration and cloud resource allocation.
• A fuzzy set of computational resources for edge computing nodes was constructed, considering CVaR. Then, based on the theory of Lagrange duality, the model is transformed into a semidefinite programming form, and the RaDROO algorithm is proposed to solve the task offloading problem with distributional robustness under risk awareness.
• We conducted simulation experiments from two aspects. On one hand, we adjusted certain parameters of the proposed model to obtain the optimal values for those parameters. On the other hand, we fixed the parameters and compared them with the state-of-the-art algorithms in different computation and network environments. The experimental results demonstrate that our proposed model and algorithm have better results than the state-of-the-art methods in terms of usability, robustness, and risk.

Table 1 shows the superiority of our algorithm compared with relevant literatures.

The remainder of this paper is organized as follows. Section 2 provides a brief introduction to SAGIN and computational task offloading. Section 3 describes the network architecture and proposes a computation task offloading model under uncertain computation environments. Section 4 presents the RaDROO algorithm for obtaining optimal task offloading decisions and resource allocation strategies. Section 5 provides the performance evaluation and analysis. Finally, Section 6 concludes the work and presents future directions for research.

## 2. Related Work

In this section, we first introduce the SAGIN architecture. We then review the traditional computation task offloading, computation task offloading under uncertain network and computation environments, and risk-aware computation offloading, respectively.

### 2.1. SAGIN Architecture

SAGIN refers to the integration and synergy of systems from multiple domains—space, air, and ground—to form a wide coverage area and a high-speed network that enables more efficient communication and data exchange. It is a proposed solution to address the growing demand for enhanced communication and information-sharing capabilities. SAGIN requires the use of various technical means, such as satellite communications, unmanned aerial vehicles, and ground sensors, to enable collaborative operations and data sharing between different platforms. By sharing data and information in real time, situational awareness can be improved, decision-making can be optimized, and mission effectiveness can be enhanced.

Over time, the SAGIN architecture has rapidly evolved, and various projects, such as the global information grid (GIG), have been proposed and widely deployed [16]. Liu et al. [3] described the communication network design and resource allocation algorithm of SAGIN in a high-dimensional network environment. Likewise, SAGIN can be invoked in MEC environments and exhibits excellent performance in unique environments, including deserts and disaster scenarios. Yu et al. [17] considered the fine-grained offloading problem and caching problem and proposed the SAGIN framework, which supports edge computing.

The impact of SAGIN on computing task offloading is multifaceted and can be summarized as follows:

− Cross-platform efficiency: Through the integration and collaborative operations achieved by SAGIN, different tasks can be offloaded and migrated between different platforms, thereby improving the efficiency of task execution.
− Increased flexibility through edge–cloud collaboration: In the SAGIN network, tasks can be dynamically allocated and scheduled, allowing them to be offloaded to the most suitable platforms based on their computational requirements. This improves resource utilization.

Overall, SAGIN has a positive impact on the field of task offloading, as it can improve task execution efficiency, safety, flexibility, and coverage. An ECN built on the SAGIN framework will be more able to fully exploit its capabilities by consolidating available resources to provide computing resources for tasks within this network environment.

### 2.2. Traditional Computation Offloading

First of all, a class of existing task offloading optimization algorithms in practical applications focuses on task dependency. Yuan et al. [18] focused on a dependent task assignment problem over multiple mobile terminal devices (MTDs). In [19], an efficient partitioned search method was implemented to obtain optimal solutions for task offloading policies and resource allocation under task-dependent models. Moreover, without considering task dependency, determining whether to offload tasks to edge computing nodes (ECNs) and how to allocate computing resources in ECNs based on the performance requirements for computing offloads are hot topics among current researchers. The three mainstream research directions under this scope are as follows. The first one is to explore task offloading decision schemes that minimize task execution delay [20,21]. Xiao et al. [22] designed a heat prediction method to analyze the dynamics of urban heat zones, aided by the design of a non-cooperative game-theoretic strategy selection based on regret-matching to achieve the minimum time delay. Dai, Y. et al. [23] proposed a JSCO algorithm to search for the solution to the optimization problem in a distributed manner with less overhead, using the integrated task processing delay as the performance metric. In [13], Farhangi, E.et al. proposed a novel offload approach, OAMC, which takes into account the dynamic changes of mobile applications. This approach aims to reduce the migration number and overall data movement while minimizing the turnaround time of mobile applications. Secondly, plenty of studies [24,25,26,27] focus on obtaining task offloading decisions in edge computing networks that minimize energy consumption, which is affected by the amount of offloading computations, the MTD-MEC distance, channel conditions, application type, compression efficiency, *etc.* Hmimz et al. [26] jointly considered the priority of certain MTDs and aimed to minimize overall power consumption. Third, the authors of [28,29] assumed that the system can gain a certain amount of revenue by completing the task, and the goal is to find the offloading decision that maximizes the revenue. Samanta et al. [30] synthetically explored delay-sensitive and delay-tolerant edge services while ensuring the maximization of quality of experience (QoE) over an extended period using the Lyapunov method.

In addition to the challenges commonly addressed by traditional optimization methods, some researchers [31] have chosen to apply intelligent optimization algorithms to determine the optimal unloading decision. In [27], the authors developed a new adaptive inertia weight-based particle swarm optimization (NAIWPSO) algorithm to minimize the energy consumption of MTDs while considering the channel constraint conditions during task offloading. Li et al. [32] proposed an algorithm, named EIPSO, which is based on a particle swarm optimization-based algorithm. Tout et al. [33] designed a multi-objective intelligent optimization algorithm based on the genetic algorithm.

Machine learning and deep learning algorithms are now extraordinarily prevalent in the research field. Maleki, E. F. et al. [13] applied a machine learning (ML) algorithm called matrix complementation to develop two novel offloading methods, S-OAMC and G-OAMC, which enhance scalability and identify offloading decisions with low turnaround times. In [34], various machine learning techniques for communication, network, and security components of future 6G environments in vehicular networks were profiled, and methods and directions for implementing artificial intelligence in future 6G vehicular networks were envisioned. However, machine learning algorithms typically require significant amounts of training data and cannot be directly computed. They rely on training with large datasets to learn patterns and make predictions. Additionally, machine learning algorithms are sensitive to changes in the environment and may require retraining or adaptation to maintain their performance.

### 2.3. Uncertain-Aware Computation Offloading

The above research scenarios assume that controllers have access to accurate computing resources and channel information, either statically or dynamically. Nevertheless, in application environments, MECNs are transient, which can considerably impact resource utilization and user QoE. In [4,7], the authors investigated robust task offloading that is tolerant to failures in offloading scenarios and can effectively overcome this. In [8], the offloading of robust tasks that can tolerate server failures was considered to achieve improved application responsiveness. To counter the inaccuracy of channel measurements, the authors of [9] designed a robust offloading strategy for channel information estimation errors. Facing the connection instability between MTDs and small clouds, the authors of [7] proposed a robust computation offloading strategy with failure recovery.

In contrast to the mentioned robust optimization approach, which trades off significant device performance to ensure robustness, stochastic optimization (SO) is employed. SO studies in mobile edge computing (MEC) take into account the probability distributions of channel resources and the random variations of computational resources in edge computing nodes (ECNs). The authors of [6] proposed a two-stage SO to tackle the challenge of uncertain dynamic environments.

### 2.4. Risk-Aware Computation Offloading

Unlike the uncertain or undefined parameter scenarios described above, risk considerations refer to unexpected conditions that occur during system operation. Bai et al. [12] solved the risk-aware computation offloading (RCO) problem by considering the use of the Bayesian Stackelberg game to rationally disperse the nodes for task offloading when the server was attacked by outside miscreants. Apostolopoulos et al. [35] considered the uncertainty in MEC server computational resources as well as storage space under heterogeneous networks. Schultz, R.et al. [15] explained the risk consideration in stochastic programming, i.e., the mean risk, and also gave the expression formula for CVaR. With CVaR, Pan et al. [36] considered the availability of the MEC system in the case of a possible link failure. Mean-CVaR is commonly deployed in finance. Zhang et al. [14] studied portfolio selections under return fuzzy set conditions, considering a downside risk measure to reduce investment risk.

## 3. Network Architecture and System Model

In this section, we elaborate on the SAGIN architecture and channel models. Based on the compute, caching, and communication (3C) requirements, we developed an optimization model for making computing offload decisions with the objective of minimizing costs.

### 3.1. SAGIN Architecture and Channel Models

SAGIN is a novel network structure that integrates multiple dimensions. It adopts different protocols and occupies different frequency bands for establishing communications between different dimensions [3]. We selected the rational frequency band for communications by taking into account the distance between ECNs, the influence of current environmental factors on the link, the free-space path loss, tropospheric attenuation, and other factors.

**Figure 1:**
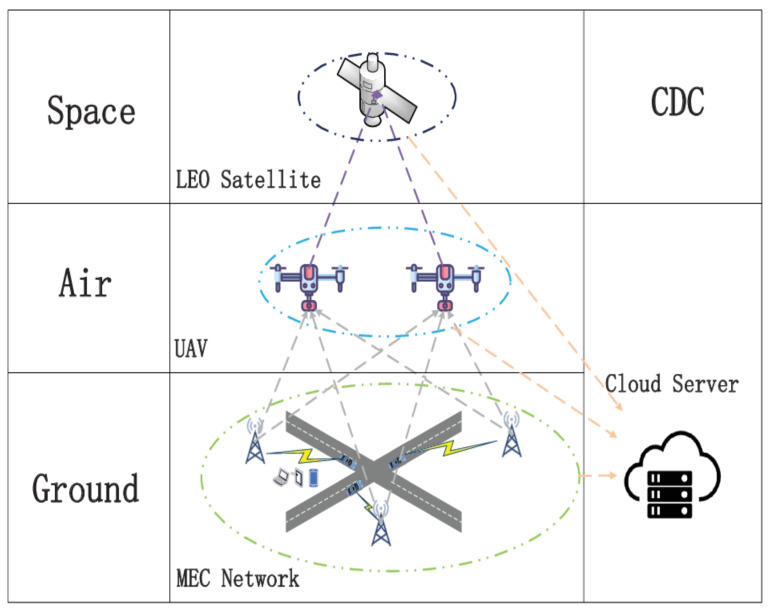
SAGIN model.

In the study, tasks were uploaded to the nearest BSs from MTDs by default. As shown in Figure 1, the SAGIN architecture consists of five components: the MTD segment, ground segment, satellite segment, aerial segment, and cloud data center (CDC) segment. Let B={1,2,⋯,B} be used as the set of BSs. Furthermore, UAVs are deployed as an extension of the ground BS process, serving as representatives of the aerial segments. The set of UAVs is denoted by U={1,2,⋯,U}. The satellite segment consists of LEO satellites. Due to the utilization of higher communication frequency bands, satellites possess lower propagation delay and free-space attenuation [37]. We consider the displacement of satellites relative to the ground during the task processing time slots to be negligible.

The SAGIN system provides three task offload strategies based on the task information uploaded by MTDs: Firstly, selecting a BS that possesses sufficient computing resources to calculate the task; secondly, applying for a UAV offload and computing the task; thirdly, selecting a UAV as an intermediary and applying an LEO satellite to offload and compute the task. As a multi-dimensional 6G network, SAGIN integrates several network segments and uses communication protocols to achieve high reliability and high-throughput data transmission [38].

The maximum achievable transmission rate K [bits/s] for the tasks in the channel can be expressed as follows:

(1) $$\left\{\begin{array}{c} K=B\log_{2}\left(1+\frac{P\times 10^{-[L_{P}/10]}}{Noise}\right),\\ L_{p}=32.45+20\mathrm{lg}d\left(km\right)+20\mathrm{lg}f\left(MHz\right). \end{array}\right.$$

Within the above equation, $B\left(HZ\right)$, *P*, and Noise represent the channel bandwidth, average signal power, and noise power; $L_{p}$, *d*, and *f* represent the propagation loss in free space, the distance between ECNs, and the signal frequency, respectively.

### 3.2. Computation Task Offloading Model

We assume that there are *n* latency-sensitivity and computation-intensive tasks offloaded to nearby BSs, where the set of tasks is denoted as N={1,2,⋯,N}. Moreover, each task is characterized by certain parameters, denoted as $\left(n_{i}^{up},n_{i}^{comp},n_{i}^{down}\right),i\in N$, where $n^{up}$ denotes the size of the input and uploaded data; $n^{comp}$ denotes the number of CPU cycles required to process the data; $n^{down}$ denotes the size of the processed and downloaded data. In this research, the objective was to study the 3C and total costs of tasks while maintaining the atomicity of the tasks, i.e., each task could only be served by one processing ECN.

In our proposed SAGIN system, there are four offload destinations: BSs, UAVs, LEO satellites, and CDC. We assume that $x^{BS}$, $x^{UAV}$, and $x^{LEO}$ are three binary parameters, where $x_{ib}^{BS}$ denotes whether task *i* selects BS *b* as the offload target, $x_{iu}^{UAV}$ denotes whether task *i* selects UAV *u* as the offload target, and $x_{is}^{LEO}$ denotes whether task *i* selects the LEO satellite *s* as the offload target.

(2) $$\sum_{b=1}^{B}x_{ib}^{BS}+\sum_{u=1}^{U}x_{iu}^{UAV}+\sum_{s=1}^{S}x_{is}^{LEO}=1,\forall i\in N$$

(3) $$x_{ib}^{BS},x_{iu}^{UAV},x_{is}^{LEO}\in \left\{0,1\right\},b\in B,u\in U,s\in S$$

The above equation indicates that each task would be offloaded to one ECN in the SAGIN architecture. $x_{ib}^{BS}=1$ if task $n_{i}$ is offloaded to BS *b* and $x_{ib}^{BS}=0$ otherwise; $x_{iu}^{UAV}=1$ if task $n_{i}$ is offloaded to UAV *u* and $x_{iu}^{UAV}=0$ otherwise; moreover, $x_{is}^{LEO}=1$ if task $n_{i}$ is offloaded to LEO satellite *s* and $x_{is}^{LEO}=0$ otherwise. These three different computing units own different computing power and storage resources. $f_{ib}^{BS}$, $f_{iu}^{UAV}$, and $f_{is}^{SAT}$ denote the number of computing resources requested by task *i* per second from BSs, UAVs, and LEO satellites, respectively.

We assume that tasks are uploaded to the nearest base station first and then propagate them from the base station to the target computing nodes. Since the propagation delay of tasks directly to edge computing nodes is much greater than the upload delay of tasks to the nearest base station, we will ignore the upload delay of tasks.

The time taken by a task to be uploaded and downloaded between ECNs and the latency taken to calculate the task can be expressed as follows:

(4) $$\begin{array}{ccc} T^{up} & =\frac{n^{up}}{k^{up}}=\frac{n^{up}}{B\log_{2}\left(1+\frac{P\times 10^{-[L_{P}/10]}}{N}\right)} & \end{array}$$

(5) $$\begin{array}{ccc} T^{down} & =\frac{n^{down}}{k^{down}}=\frac{n^{down}}{B\log_{2}\left(1+\frac{P\times 10^{-[L_{P}/10]}}{N}\right)} & \end{array}$$

(6) $$\begin{array}{cc} T^{comp} & =L^{time}-T^{up}-T^{down} \end{array}$$

where $T^{comp}$ is the time to execute the task, $T^{up}$ and $T^{down}$ represent the time of uploading and downloading the task message.

The overall latency of the tasks offloaded to the BSs comprises the transfer delay to the target BS, the computation delay of the BS, and the transfer delay of the result download.

(7) $$T_{ib}^{BS}=T_{ib}^{up}+T_{ib}^{comp}+T_{ib}^{down},i\in N$$

One option is to upload tasks to the UAVs when the computing resources on the ground are insufficient to handle the high influx of tasks.

(8) $$T_{iu}^{UAV}=T_{iu}^{up}+T_{iu}^{comp}+T_{iu}^{down},i\in N$$

The task process changes with the calculations involved in offloading tasks to LEO satellites. Task information must be uploaded to the nearest UAV before spreading to satellites. After the task processing is completed, the data are returned to the original path. The total latency taken to offload the tasks to an LEO satellite can be expressed as follows:

(9) $$T_{is}^{LEO}=T_{iu}^{up}+T_{is}^{up}+T_{s}^{comp}+T_{iu}^{down}+T_{ib}^{down}$$

where $T_{ibu}^{up}$, $T_{ius}^{up}$, $T_{isu}^{down}$, $T_{iub}^{down}$ and $T_{is}^{comp}$ represent the uploading latency of task *i* from BSs to UAVs, from UAVs to LEO satellites, the downloading latency from LEO satellites to UAVs, from UAVs to BSs, and the computation delay of task *i*, respectively.

For this paper, we ensured that the task was completed within the time delay constraints. Therefore, to allocate computing resources rationally, the node only provides the lowest possible computational resources, $f_{i}=n_{i}^{comp}\;/\left(L_{i}^{T}-T^{trans}\right)$, where $T_{i}^{trans}$ represents the time taken for task *i* to be transmitted over the link.

There is an upper bound on the total amount of computing resources an ECN can have. As a result, the combined computational resources required for the tasks assigned to a node should not exceed the upper limit.

(10) $$\begin{array}{cc} \sum_{i=1}^{n}f_{i}x_{ib}^{BS} & \leq L_{b}^{Bcomp},\forall b\in B, \end{array}$$

(11) $$\begin{array}{cc} \sum_{i=1}^{n}f_{i}x_{iu}^{UAV} & \leq L_{u}^{Ucomp},\forall u\in U, \end{array}$$

(12) $$\begin{array}{cc} \sum_{i=1}^{n}f_{i}x_{is}^{LEO} & \leq L_{s}^{Lcomp},\forall s\in S, \end{array}$$

We consider caching in 3C, where each ECN has its individual storage limit.

(13) $$\begin{array}{cc} \sum_{i=1}^{N}x_{ib}^{BS}n_{i}^{up} & \leq L_{b}^{Bcache},\forall b\in B \end{array}$$

(14) $$\begin{array}{cc} \sum_{i=1}^{N}x_{iu}^{UAV}n_{i}^{up} & \leq L_{u}^{Ucache},\forall u\in U \end{array}$$

(15) $$\begin{array}{cc} \sum_{i=1}^{N}x_{is}^{LEO}n_{i}^{up} & \leq L_{s}^{Lcache},\forall s\in S \end{array}$$

$L_{b}^{Bcache}$, $L_{b}^{Ucache}$, and $L_{b}^{Lcache}$ represent the cache upper bound of the BS *b*, UAV *u*, and LEO satellite *s*, separately.

The cost for task offloading is closely tied to the server type and the number of required computing resources. In this paper, we focus on solving computationally intensive and time-delay-sensitive tasks. Therefore, minimizing the offload cost by selecting an appropriate offloading target is crucial. Hence,

(16) $$\begin{array}{cc} C_{i}= & \sum_{b=1}^{B}\left(\delta_{b}^{BS}\times n_{i}^{comp}\times x_{ib}^{BS}\right)\\ \; & +\sum_{u=1}^{U}\left(\delta_{u}^{UAV}\times n_{i}^{comp}\times x_{iu}^{UAV}\right)\\ \; & +\sum_{s=1}^{S}\left(\delta_{s}^{LEO}\times n_{i}^{comp}\times x_{is}^{LEO}\right),i\in N \end{array}$$

(17) $$C^{all}=\sum_{n=1}^{N}C_{n}$$

In the above equation, $C_{i}$ denotes the final cost of task *i*; $C^{ALL}$ denotes the cost for accomplishing whole tasks; $\delta_{b}^{BS}$, $\delta_{b}^{UAV}$, and $\delta_{b}^{LEO}$ represent the ratio between the fees and the data size that will be processed by BSs, UAVs, and LEO satellites, respectively

(18) $$\begin{array}{cc} \; & \min_{x_{ib}^{BS},x_{iu}^{UAV},x_{is}^{LEO}}C^{all}\\ s.t.\; & \left(2),\;(3),\;(10),\;(11),\;(12),\;(13),\;(14),\;(15\right) \end{array}$$

### 3.3. Computing Resources Using a Fussy Set Model

Realistically, the computational resources of each node can be greatly influenced by external factors. Qu et al. [4] considered the resource uncertainty of MECN in a traditional environment. In this paper, to account for the computing resource uncertainty of ECNs, we propose a realistic scenario-based set with a definite probability distribution to represent the possible range of uncertain resources.

Based on a fuzzy set of random parameters ξ, we will make the one-stage offloading decision and the two-stage cloud resource request.

Delage, E. et al. [39] considered a set of uncertainties with specific boundaries and moments, derived from real-world data, i.e.,

(19) $$\begin{array}{cc} & D\left(\xi ,\sum ,\mu_{0},\gamma_{1},\gamma_{2}\right)\\ \; & =\left\{P\subseteq F:\begin{array}{c} P\left\{\xi \in M\right\}=1\\ {\left(E\left[\xi \right]-\mu_{0}\right)}^{T}\sum^{-1}\left(E\left[\xi \right]-\mu_{0}\right)⩽\gamma_{1}\\ E\left[\left(\xi -\mu_{0}\right){\left(\xi -\mu_{0}\right)}^{T}\right]⩽\gamma_{2}\sum \end{array}\right\} \end{array}$$

where parameters $\gamma_{1}$ and $\gamma_{2}$, based on historical data, are utilized to control the size of the ambiguity set and the conservatism of optimal solutions. M is the closed convex set representing the support of the currently known random variable ξ, and ∑ and $\mu_{0}$ are, respectively, its corresponding first-order and second-order moments. In contrast, F is the set of all probability measures on the measurable space ($R^{m}$, B), where B represents the Borel β-algebra on $R^{m}$.

The first constraint indicates that the probability density sum of ξ is 1 over $M^{\xi }$; the second constraint assumes that the mean of ξ lies on an ellipsoid of size $\gamma_{1}$ centered at $\mu_{0}$; the third constraint assumes that the covariance matrix lies in a positive semi-positive definite cone bounded by matrix inequalities.

## 4. Risk-Aware Distributionally Robust Optimization Task Offloading Algorithm Design

In this section, we consider fuzzy sets and CVaR to address the uncertainty of computational resources in edge computing nodes. In the first stage, we consider the computational resources required for performing computations on the currently available computing nodes. In the second stage, we take into account the cost associated with additional computational resources that may need to be requested from the cloud due to the uncertainty of node computational resources. As the computational resources are presented in the form of fuzzy sets, the second stage is represented by taking the mean value in the RaDROO algorithm. Additionally, to consider CVaR, we use lambda as a weight and select the mean value of the poorer part in the fuzzy set to obtain a more robust offloading solution.

### 4.1. Network Architecture and System Model

In this section, we consider the transformation of the original problem into a two-stage DRO programming model with a mean-CVaR recourse function, namely a risk-aware DRO model.

Above all, we express (18) in terms of matrix form variables and parameters. We consider the set D=B∪U∪S and M=B+U+S. The formulated problem is written as follows:

(20) $$\begin{array}{cc} \; & \min_{X}\;\;{N^{comp}}^{T}\times X\times \delta \end{array}$$

(20a) $$\begin{array}{cc} & s.t.\;X\times O_{m}\;=\;O_{n}, \end{array}$$

(20b) $$\begin{array}{cc} & \;\;\;\;\;X^{T}\times N^{up}\leq L^{cache}, \end{array}$$

(20c) $$\begin{array}{cc} & \;\;\;\;\;\;\;\;\;\;\;\;\;\;\;\;\;\;\;\;\;\;\;\;\;\;\;\;\;\;\;\;\;\;\;\;\;\;\;\;X^{T}\times F\leq L^{comp},\\ & \;\;\;\;\;\;\;\;\;\;\;\;\;\;\;\;\;\;\;\;\;\;\;\;\;\;\;\;\;\;\;\;\;\;\;\;\;\;\;\;X\in R^{N\times M},\delta \in R^{N},N^{comp}\in R^{M},N^{up}\in R^{N}, \end{array}$$

(20d) $$\begin{array}{cc} & \;\;\;\;\;\;\;\;\;\;\;\;\;\;\;\;\;\;\;\;\;\;\;\;\;f\in R^{N},L^{cache}\in R^{M},L^{comp}\in R^{M} \end{array}$$

where the offloading strategy matrix variable $X:={\left\{x_{nm}\right\}}_{n\in N,m\in D}$, the upper bound of the computing resource vector parameter $L^{comp}:={\left\{L_{m}^{comp}\right\}}_{m\in D}$, the upper bound of the cache ceiling vector parameter $L^{cache}:={\left\{L_{m}^{cache}\right\}}_{m\in D}$, the computing resource vector parameter $F:={\left\{f_{i}\right\}}_{i\in N}$, the ratio between the cost and the data size vector parameter $\delta :={\left\{\delta_{i}\right\}}_{i\in N}$, and the all-ones vector parameter is *O*.

Thus, given the uncertainty of the parameter $L^{comp}$, a two-stage offloading strategy model is created using the distribution set proposed above. We constructed a fuzzy set based on the historical data of the computing resources of the offload nodes, expressed as follows: $\xi ={\tilde{L}}^{comp}$.

(21) $$\begin{array}{cc} \min_{X}\;\;\;\; & {N^{comp}}^{T}\times X\times \delta +E_{\xi }\left[\vartheta \left(X,\xi \right)\right],\\ s.t. & \left((20a)-(20d)\right), \end{array}$$

(21a) $$\begin{array}{cc} & \;\;\;\left\{P\subseteq F^{\xi }:\begin{array}{c} P\left\{\xi \in M^{\xi }\right\}=1\\ {\left(E\left[\xi \right]-\mu_{0}\right)}^{T}\sum^{-1}\left(E\left[\xi \right]-\mu_{0}\right)\leq \gamma_{1}\\ E\left[\left(\xi -\mu_{0}\right){\left(\xi -\mu_{0}\right)}^{T}\right]\leq \gamma_{2}\sum \end{array}\right\} \end{array}$$

(21b) $$\begin{array}{cc} with\;\; & \vartheta \left(X,\xi \right)=\left\{\begin{array}{c} \min_{Y}\;\;\varphi^{T}\times Y,\\ {\left(X⊗F^{req}\right)}^{T}\times O_{n}⩽\xi +Y,\\ 0\leq Y, \end{array}\right. \end{array}$$

$\vartheta \left(X,\xi \right)$ represent the additional cost of uploading tasks to the cloud when the real-time computing resources change in a phase of decision task offloading and are insufficient to meet demand. Vector φ indicates the ratio of additional costs to required computational power.

Instead, we further consider the mean-CVaR (conditional value at risk) criterion in the model, which can be expressed in the following form:

(22a)VaRα(ζ)=infv∈R:Fv≥αCVaRα(ζ)=E{ζ|ζ≥VaRα(ζ)}(22b)=minv∈R{v+11−αE[(ζ−v)+]}

where F is the cumulative distribution function of the function used to solve the random variable ζ, and a is the given confidence level. ${\left(a\right)}_{+}$ means $\max\left(a,0\right)$.

This model gives the set of possible distributions $M^{\xi }$ for the unknown stochastic parameter ξ, while ensuring its robustness through a mean-CVaR approach with a two-stage minimization of fees. The following formulation is made using the min–max theory:

(23) $$\begin{array}{cc} \; & \min_{X}\;\;h\left(x\right)+\underset{\xi }{\sup}\left\{\left(1-\lambda \right)E\left[\vartheta (x,\xi )\right]+\lambda CVaR\left(\vartheta \left(x,\xi \right)\right)\right\}\\ & with\;h\left(x\right)={N^{comp}}^{T}\times X\times \delta ,\\ & s.t.\;(20a),\;(20b),\;(20c),\;(20d),\;(21a),\;(21b), \end{array}$$

where λ indicates the trade-off coefficient of conditional risk considered in the objective function, with 1≥λ≥0.

### 4.2. Transform from DRO to SDP

We reconstruct the original problem by considering the uncertainty of computational resources and acknowledging the challenge of directly addressing the DRO problem. To solve this, we employ the Lagrangian dual to transform it into a semi-definite programming (SDP) problem.

Problem (23) presents a DRO problem. Below, we further analyze the target formula and convert it into a more explicit form.

(24) $$\begin{array}{cc} \; & \underset{\xi }{\sup}\left\{\left(1-\lambda \right)E[\vartheta (x,\xi )]+\lambda CVaR(\vartheta (x,\xi ))\right\}\\ = & \underset{\xi }{\sup}\left\{\;\min_{v\in R}\left\{\lambda v+\left(1-\lambda \right)E\left[\vartheta \left(x,\xi \right)\right]+\frac{\lambda }{1-\alpha }E\left[{\left(\vartheta \left(x,\xi \right)-v\right)}_{+}\right]\right\}\right\}\\ = & \min_{v\in R}\left\{\lambda v+\underset{\xi }{\sup}\left\{\;E\left[\left(1-\lambda \right)\vartheta \left(x,\xi \right)+\frac{\lambda }{1-\alpha }{\left(\vartheta \left(x,\xi \right)-v\right)}_{+}\right]\right\}\right\} \end{array}$$

The ambiguous distribution set of the random parameter cannot be used directly for the solution, and we need to convert it into the common inequality form. Consider only the latter half, which involves finding the optimal value in relation to the random parameter ξ. Instead of the original distribution set form, we transform it into the following semi-infinite conic linear problem form, expressed as:

(25) $$\begin{array}{cccc} \; & \; & \underset{\xi }{\sup} & \int_{M}\left[\left(1-\lambda \right)\vartheta \left(x,\xi \right)+\frac{\lambda }{1-\alpha }{\left(\vartheta (x,\xi )-v\right)}_{+}\right]\;\;\;d\;f\left(\xi \right) \end{array}$$

(25a) $$\begin{array}{cccc} \; & \; & s.t. & \int_{M}d\;f\left(\xi \right)=1, \end{array}$$

(25b) $$\begin{array}{cccc} \; & \; & & \;\;\;\;\;\;\;\;\;\;\;\;\;\;\;\;\;\;\;\;\;\;\;\;\;\;\;\;\;\;\;\int_{M}\left(\xi -\mu_{0}\right){\left(\xi -\mu_{0}\right)}^{T}d\;f\left(\xi \right)≼\gamma_{2}\sum , \end{array}$$

(25c) $$\begin{array}{cccc} \; & \; & & \;\;\;\;\;\;\;\;\;\;\;\;\;\;\;\;\;\;\;\;\;\;\;\;\;\;\;\;\;\;\;\int_{M}\left[\begin{array}{cc} \sum & \xi -\mu_{0}\\ {\left(\xi -\mu_{0}\right)}^{T} & \gamma_{1} \end{array}\right]d\;f\left(\xi \right)≽0, \end{array}$$

(25d) $$\begin{array}{cccc} \; & \; & & \xi \in M^{\xi }, \end{array}$$

Secondly, we utilize the duality theory to convert it into an SDP problem. Consider the dual of problem (25):

$$\begin{array}{cc} & \left(Dual\right):\; \end{array}$$

(26) $$\begin{array}{cc} & \underset{r,H,\left[\begin{array}{cc} Z & z\\ z^{T} & \hat{z} \end{array}\right]}{\inf}r+\langle H,\gamma_{2}\sum -\mu_{0}\mu_{0}^{T}\rangle +\langle Z,\sum \rangle +\hat{z}\gamma_{1}-2z^{T}\mu_{0} \end{array}\;$$

(26a) $$\begin{array}{cc} & s.t.\;u\left(x,\xi \right)-r-\xi H\xi^{T}+2\xi^{T}H\mu_{0}+2z^{T}\xi \leq 0,\xi \in M^{\xi }, \end{array}$$

(26b) $$\begin{array}{cc} & \;r\in R, \end{array}\;$$

(26c) $$\begin{array}{cc} & \;H≽0, \end{array}\;$$

(26d) $$\begin{array}{c} \;\;\;\;\left[\begin{array}{cc} Z & z\\ z^{T} & \hat{z} \end{array}\right]≽0,\\ with\;\;\;u\left(x,\xi \right)=\left(1-\lambda \right)\vartheta \left(x,\xi \right)+\frac{\lambda }{1-\alpha }{\left(\vartheta (x,\xi )-v\right)}_{+}\; \end{array}$$

where *r*, *H*, and $\left[\begin{array}{cc} Z & z\\ z^{T} & \hat{z} \end{array}\right]$ are, correspondingly, the dual variables of constraints (25a), (25b), and (25c). $\langle a,b\rangle$ represents Trace(AB).

Due to the inclusion of ${\left(\right)}_{+}$, the inequality constraint (26a) has to be processed as follows:

$$\begin{array}{ccc} & r+\xi H\xi^{T}-2\xi^{T}H\mu_{0}-2z^{T}\xi & \\ ⩾ & \left(1-\lambda \right)\vartheta \left(x,\xi \right)+\frac{\lambda }{1-\alpha }{\left(\vartheta \left(x,\xi \right)-v\right)}_{+} \end{array}$$

When the random parameter ξ appears in the constraint, it cannot be directly substituted into constraint (26a). Therefore, it is necessary to consider the dual of problem ϑ(x,ξ), where the random parameters are transferred into the objective function. Another problem arises, which is the coupling of variables between the dual variable *q* and variable *Y*. The constraint on the dual variable *q* confines it to a boxed region, as indicated by the constraint.

$$\begin{array}{ccc} \; & \left(Dual\right):\; & \end{array}$$

(27) $$\begin{array}{ccc} \; & \vartheta^{\prime }\left(x,\xi \right)=\max_{q,p}q^{T}\left({\left(X⊗F^{req}\right)}^{T}O_{N}-\xi \right), & \end{array}$$

(27a) $$\begin{array}{cc} s.t.\;\varphi -p⩾q⩾0, & \; \end{array}$$

(27b) $$\begin{array}{cc} \;p⩾0, & \; \end{array}$$

Responding to constraint (26a), taking its extreme value points, each point corresponds to two corresponding semi-definite matrix constraints. We transformed (26a) into a clearer and more concise form.

$$\begin{array}{c} \left\{\begin{array}{cc} \; & r+\xi H\xi^{T}-2\xi^{T}H\mu_{0}-2z^{T}\xi \\ \; & ⩾\left(1-\lambda \right)\vartheta^{\prime }\left(x,\xi \right)+\frac{\lambda }{1-\alpha }\left(\vartheta^{\prime }\left(x,\xi \right)-v\right)\\ \; & =\left(1+\frac{\lambda \alpha }{1-\alpha }\right)\vartheta^{\prime }\left(x,\xi \right)-\frac{\lambda v}{1-\alpha }\\ \; & r+\xi H\xi^{T}-2\xi^{T}H\mu_{0}-2z^{T}\xi ⩾\left(1-\lambda \right)\vartheta^{\prime }\left(x,\xi \right) \end{array}\right. \end{array}$$

In the above constraints, we denote $\left(1+\frac{\lambda \alpha }{1-\alpha }\right)$ as ϕ and transform them into the following two semi-definite matrix constraints.

$$\begin{array}{c} \left\{\begin{array}{c} \left[\begin{array}{cc} H & \frac{1}{2}\phi q^{T}-H\mu_{0}-z\\ {\left(\frac{1}{2}\phi q-H\mu_{0}-z\right)}^{T} & r-\phi q^{T}{\left(X⊗F^{req}\right)}^{T}O_{N}+\frac{\lambda v}{1-\alpha } \end{array}\right]≽0\\ \left[\begin{array}{cc} H & \frac{1-\lambda }{2}q-H\mu_{0}-z\\ {\left(\frac{1-\lambda }{2}q-H\mu_{0}-z\right)}^{T} & r-\left(1-\lambda \right)q^{T}{\left(X⊗F^{req}\right)}^{T}O_{N} \end{array}\right]≽0 \end{array}\right. \end{array}$$

The dual problem (26) can then be rewritten as follows:

(28) $$\begin{array}{cc} & \min_{X,v,r,H,\left[\begin{array}{cc} Z & z\\ z^{T} & \hat{z} \end{array}\right]}h(x)+\lambda v+r+\langle H,\gamma_{2}\sum -\mu_{0}\mu_{0}^{T}\rangle \\ & \;+\langle Z,\sum \rangle +\hat{z}\gamma_{1}-2z^{T}\mu_{0} \end{array}$$

(28a) $$\begin{array}{cc} s.t.\;X\times O_{M}\;=\;O_{N}, & \; \end{array}$$

(28b) $$\begin{array}{cc} \;X^{T}\times N^{up}⩽L^{cache}, & \; \end{array}$$

(28c) $$\begin{array}{cc} & \;\left[\begin{array}{cc} H & \frac{1}{2}\phi q^{T}-H\mu_{0}-z\\ {\left(\frac{1}{2}\phi q-H\mu_{0}-z\right)}^{T} & r-\phi q^{T}{\left(X⊗F^{req}\right)}^{T}O_{N}+\frac{\lambda v}{1-\alpha } \end{array}\right]≽0 \end{array}$$

(28d) $$\begin{array}{cc} & \;\left[\begin{array}{cc} H & \frac{1-\lambda }{2}q-H\mu_{0}-z\\ {\left(\frac{1-\lambda }{2}q-H\mu_{0}-z\right)}^{T} & r-\left(1-\lambda \right)q^{T}{\left(X⊗F^{req}\right)}^{T}O_{N} \end{array}\right]≽0 \end{array}$$

(28e) $$\begin{array}{cc} \;\left[\begin{array}{cc} Z & z\\ z^{T} & \hat{z} \end{array}\right]≽0,H≽0,r\in R, & \; \end{array}$$

(28f) $$\begin{array}{cc} \;x_{ij}=\left\{0,1\right\},v\in R, & \; \end{array}$$

### 4.3. RADROO-MILP Algorithm

In this paper, the task offloading decision is an integer programming problem, and the additional computational resources requested from CDC follow linear programming, which makes our problem $\left(19\right)$ a MILP problem. Hence, we used the branching implicit enumeration method to obtain the optimal offloading decision based on the uncertainty of computational resources and the minimum average cost.

In the above SDP problem, the X-matrix variables, 0-1 integer variables, the Y-vector variables, and continuous variables, are included. This makes the original problem a mixed-integer linear formulation, which poses a challenge in terms of direct solvability. To simplify the problem, we relax it by treating the discrete variable *X* as a continuous variable ranging from 0 to 1, i.e., $0⩽x_{ij}⩽1,\forall x_{ij}\in X$.

In fact, the above SDP problem can be solved by using the SDPT3 solver through the CVX package in MATLAB. The original MILP problem then needs to be solved; here, we use the branching implicit enumeration method to solve it. This method is a specialized branch and bound technique for 0-1 integer problems, leveraging the fact that the variables can only take values of 0 or 1, allowing for branching for the purpose of hidden enumeration. Each task is divided into m sub-nodes, i.e., each task can only be offloaded to and completely offloaded on a single node.

Algorithm 1 presents the procedure for calculating the minimum cost of computation offloading. First of all, tasks should be sorted according to the number of CPU cycles they require, which determines the cost spent to a large degree. The solution to the relaxed LP problem is then obtained. Based on this solution, the implicit enumeration method is applied to solve the branching subproblem. During the branch and bound process, the two nodes with the highest values in the $X_{i}$ vector, representing the most probable nodes for offloading the current task, are selected at each iteration. The process involves constant branching, resulting in a gradually increasing lower bound, and constant delimitation, resulting in a gradually decreasing upper bound.
**Algorithm 1** Cost-based MILP algorithms for the DRO task offloading problem.**Input**: the convex probability distributional set $M^{\xi }$, stochastic sample space $F^{\xi }$, trade-off coefficient λ, confidence level α, ambiguous set parameters $\gamma_{1}$ and $\gamma_{2}$;**Output**: offload decision *X*, optimal cost value to satisfy distribution robustness;Solve the MILP formulation SDP problem with a confidence level of α, to obtain the optimal values of the continuous variables *X* and the optimal cost value;**for** *i* from 0 to N−1 **do**   Sort(N)   $\left[opt,\;X\right]=\max\left(X_{i}\right)$   $\left[x1_{ij},x2_{ij}\right]=max2\left(X\right)$   **if** $SDP\left(X^{\prime },x_{i,j1}^{\prime }=1\right)\;\leq \;SDP\left(X^{\prime },x_{i,j2}^{\prime }=1\right)$ **then**     $x_{i,j1}^{\prime }=1$     $min\_cost\left[i\right]=SDP\left(X^{\prime },X_{i,j1}^{\prime }=1\right)$   **else**     $x_{i,j2}^{\prime }=1$     $min\_cost\left[i\right]=SDP\left(X^{\prime },X_{i,j2}^{\prime }=1\right)$   **end if****end for****if** min_cost[N−1]!=NaN **then**   $return\;X^{\prime },\;min\_cost\left[N-1\right]$**end if**

### 4.4. Complexity Analysis

In Algorithm 1, a solver is used to solve the optimization problem. Here, the time complexity for solving the optimization problem is set to *T*, the number of unloading tasks is set to *N*, and the branch and bound method is used to traverse all unloading tasks. In the unloading target section, the process is simplified to selecting only the optimal two node locations using a cycle number. The time complexity of the proposed algorithm is O(TN).

## 5. Performance Evaluation

In this section, we evaluate the performance and effectiveness under different parameters, analyze the results of the above-proposed algorithm under different risk confidence conditions, and compare them with the traditional algorithm to highlight its superiority.

### 5.1. Simulation Setup

We accomplished the simulation on a laptop with an AMD Ryzen 7 4800H with Radeon Graphics running at 2.90 GHz. The laptop had 16GB of RAM and was running on the Windows 10 operating system. We envision a SAGIN-Cloud system where the coverage of the space layer includes both the air layer and the entire MECN. The following parameters are randomly generated within a certain range, with mean values generated according to a normal distribution.

*(1)*  *Channel State Information (CSI)*

For information transportation within ECNs, different frequency bands were borrowed between the different layers, resulting in different bandwidths and transmission speeds. Currently, the C-band is one of the most commonly used frequency bands for satellite operations, with the Ka-band being a latecomer. This paper assumes that the data transmission between BSs and UAVs occupies the C-band, while the Ka-band is occupied between UAVs and LEO satellites. The bandwidth of the C-band is 20 MHz, while the available bandwidth of the Ka-band can reach up to 3500 MHz. As for the signal frequency range, the C-band and Ka-band are 3.4–8 GHz and 26.5–36 GHz, respectively.

*(2)*  *ECNs Information*

The horizontal and vertical coordinates of the BSs on the ground and the UAVs in the air are within $\left(-1,1\right)$ km, respectively, while vertically, they are all 0. In contrast, the UAVs are located at altitudes of (0.075, 0.15) km. LEO satellites are located in space and they are at distances of (780, 800) km from the Earth’s surface. LEO satellites are located in space and they are (780, 800) km away from the surface. They are not too space-constrained, so they are deployed in a square area with four points as vertices: (−200, −200), (−200, 200), (200, −200), and (200, 200).

BSs, UAVs, and LEO satellites possess CPU process speeds of $2*10^{9}$ cycles/s, $3*10^{8}$ cycles/s, and $5*10^{9}$ cycles/s, respectively.

*(3)*  *Comparison Algorithm*

First, we propose using the most basic comparison algorithm, namely the Brute-Force algorithm.

• The Brute-Force algorithm does not take into account any uncertainty or potential computational overflow of tasks to the extent that it may eventually result in the inability to obtain an optimal solution.

Second, we compare two traditional algorithms that take parameter uncertainty into account: the RO algorithm and SO algorithm.

• In RO, only the uncertainty of computational resources of ECNs is considered and their possible worst-case scenarios are experimentally selected to ensure their robustness.
• In SO, the fuzzy set is constructed based on the historical data of the computational resources of the given ECNs; thus, we obtain their means and variances.

Third, there is another algorithm closest to our proposed one, which is the DRO algorithm; this is for obtaining the optimal solution.

• In DRO [5], the mean value in the range of the uncertainty set is obtained, and its optimal robust result is obtained by the min–max theory, which guarantees both robustness and practicality. However, there is also a drawback, namely that it is an uncertainty set constructed from historical data, which does not guarantee the stability of the uncertainty set and does not consider its risk.

Finally, we propose the RaDROO algorithm, on the basis of the traditional DRO algorithm.

• In RaDROO, in addition to what the DRO considers, it complements certain deficiencies that it possesses. It considers CVaR, choosing only the α−tail part as the benchmark, while using λ as a weight with the original part. That is to say, it selectively aggravates the proportion of the worse part of the results within the final result in order to guarantee its risk resistance.

### 5.2. Experiments

#### 5.2.1. RaDROO Algorithm

**Figure 2:**
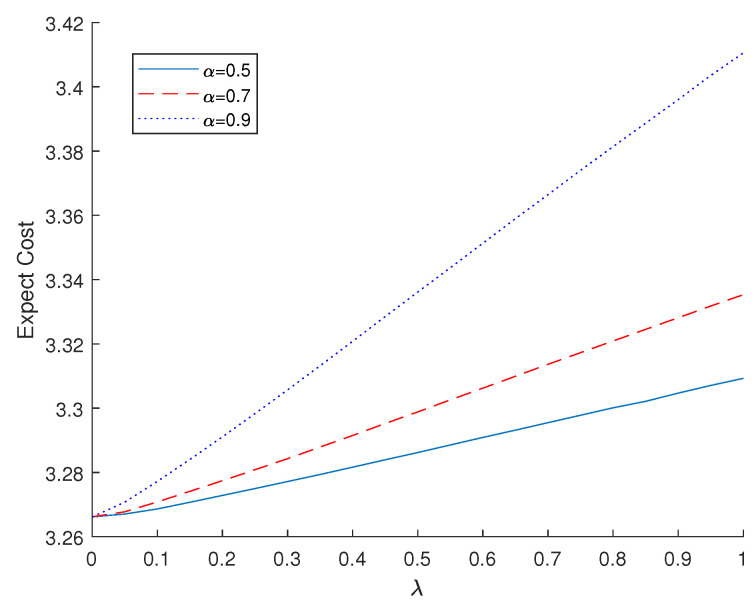
Expected costs under different α.

First, we observe the practical results of our proposed RaDROO algorithm in the context of addressing the optimization of offloading costs for edge network tasks. The optimal values of lambda in the range of (0, 1) are given as α of 0.5, 0.7, and 0.9, respectively. As illustrated in Figure 2, when lambda is 0, it means that CVaR is not considered, is weakened to a classical distribution robust problem, and the obtained results converge to a point. However, as λ increases, the risk consideration is reinforced step by step, further constraining the range of values provided within the uncertainty set. This results in a progressive increase in the amount to be spent, but at the same time, a more superior and stable optimal choice is obtained. At the same time, the larger the lambda, i.e., the larger the weight considering the CVaR value, the worse the optimal value of the required spend.

**Figure 3:**
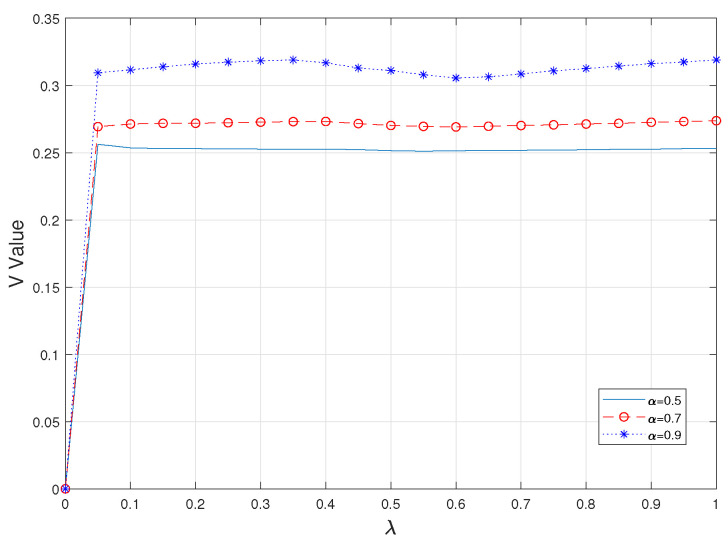
V values under different α.

**Figure 4:**
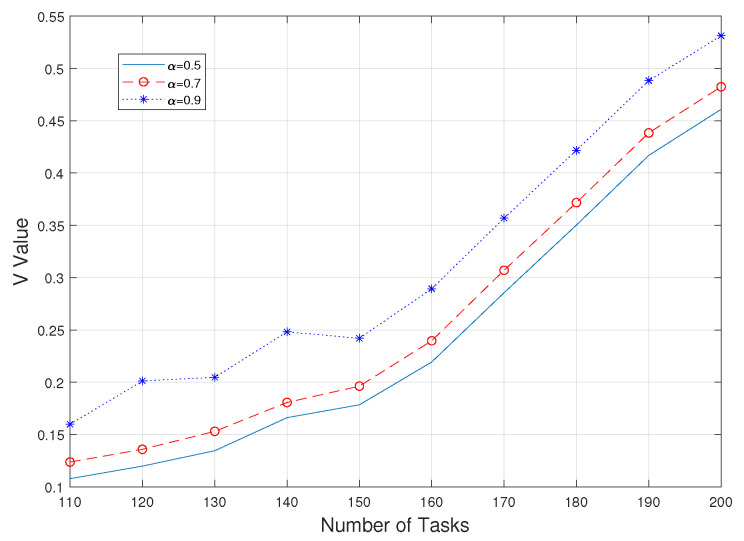
V values between 110 and 200 tasks.

Second, observing the value of V gives a more intuitive view of the degree of consideration of risk in CVaR. As shown in Figure 3 and Figure 4, it is obvious that the value of V is larger when a higher alpha value is taken, i.e., when the worse part of the fuzzy set is used as a criterion; at the same time, this value is almost unaffected by the value of λ taken. However, with a fixed value of α, as the simultaneous uploading of tasks increases, the value of V for each experiment also increases gradually and steadily, which means that the value of CVaR also increases; that is, the risk is further considered as a way to ensure the authenticity and practicality of the whole system.

#### 5.2.2. Comparison with Other Algorithms

For a comparison with other algorithms, some of the parameters of RaDROO are $\lambda \;=\;0.5,\alpha =0.5,\gamma_{1}=0,\gamma_{2}=1$.

**Figure 5:**
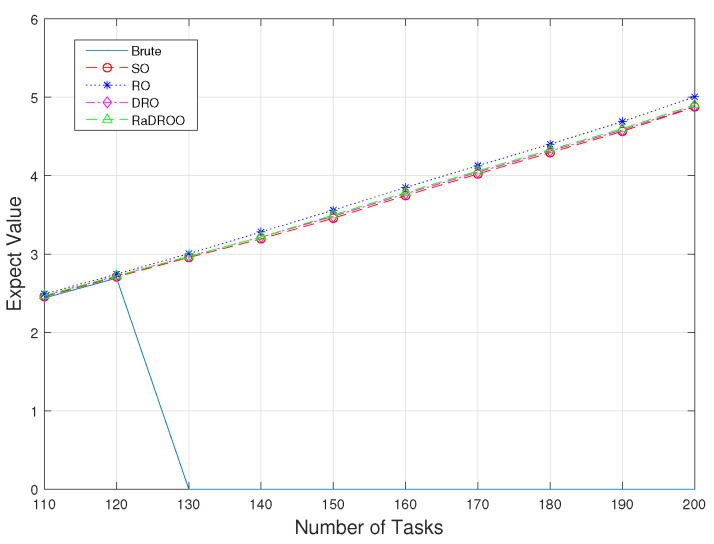
Optimal costs of the compared algorithms between 110 tasks and 200 tasks.

**Figure 6:**
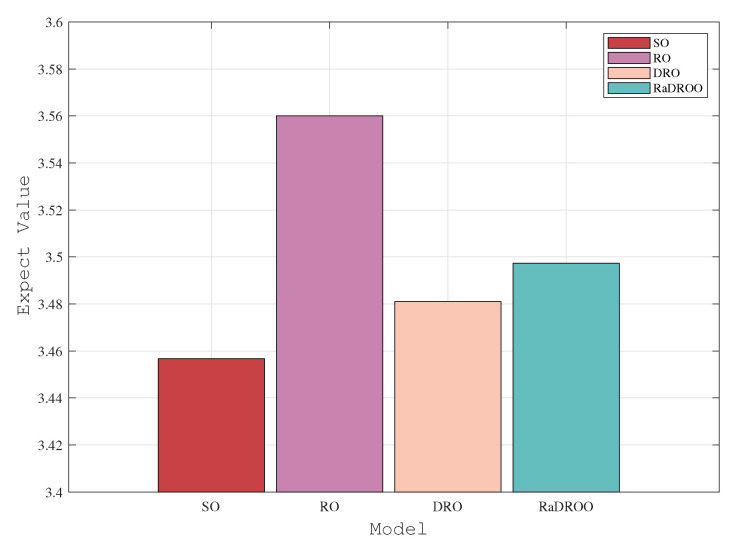
Optimal costs under 150 tasks.

**Figure 7:**
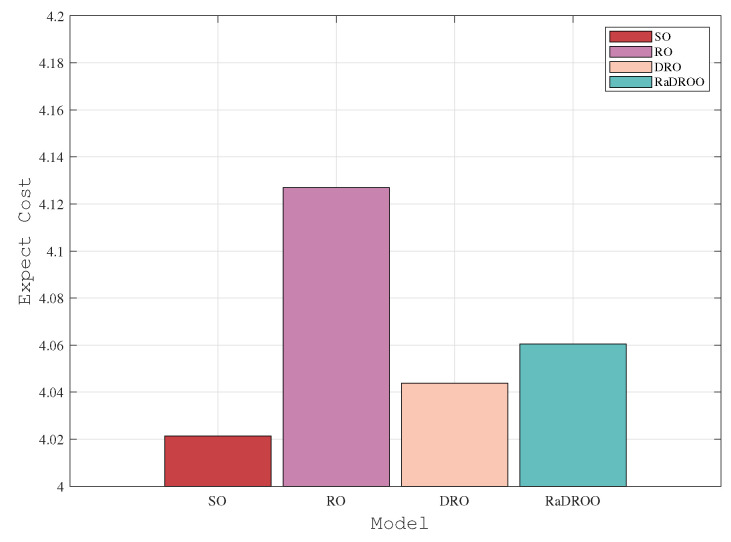
Optimal costs under 170 tasks.

The comparison between algorithms takes the obtained target value as the evaluation criterion. Figure 5 shows the optimal cost results obtained by different algorithms at the same time as the number of tasks received increases. Figure 6 and Figure 7 present a comparison of the results obtained from the four algorithms at task volumes of 150 and 170. When the number of tasks is small, and only known allocatable resources are considered, all algorithms provide better results. As the total number of tasks that may be uploaded simultaneously rises, the Brute-Force algorithm fails to obtain an optimal solution because it does not take into account the lack of computational resources. In contrast, RO, SO, DRO, and RaDROO can obtain their optimal solutions, and the optimal cost obtained increases with the increasing number of tasks.

In the two observations with 150 and 170 tasks, we can clearly see from Figure 6 and Figure 7 that the cost results obtained by the RaDROO algorithm are slightly higher than those of the traditional DRO algorithm. Obviously, as the number of tasks gradually increases, the Brute-Force algorithm that fails to consider offloading the second-stage tasks to the cloud for computations can no longer produce solutions. At the same time, the results obtained by the RaDROO algorithm and the DRO algorithm are close to those of the SO algorithm, while significantly lower than those of the RO algorithm. The SO algorithm, as it calculates the average of the fuzzy sets, is capable of obtaining excellent results, but it may not align closely with real-world scenarios. On the other hand, the RaDROO algorithm considers various factors and still manages to achieve results that are close to those of the SO algorithm, demonstrating its superiority. This indicates that the RaDROO algorithm is still capable of achieving favorable results. By incorporating CVaR to enhance risk resistance, it generates superior outcomes even under uncertain computational resource conditions.

**Figure 8:**
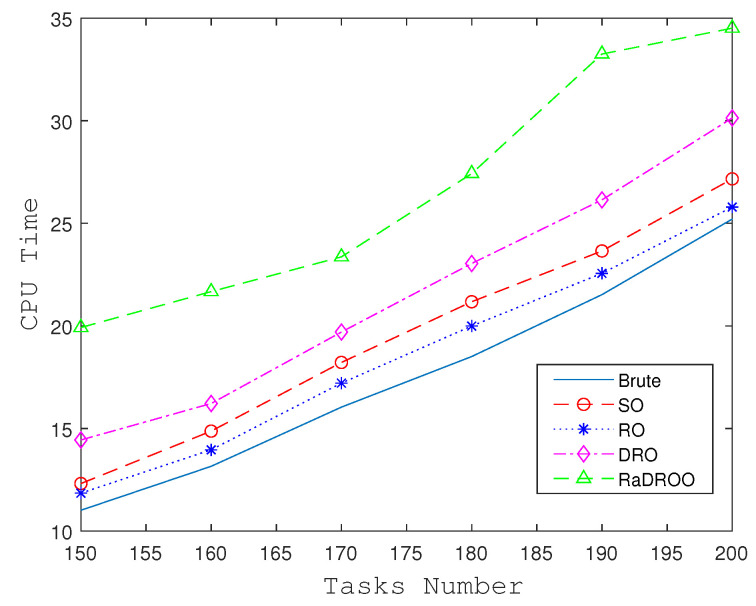
Execution time of each algorithm.

The RaDROO algorithm, although yielding better results, comes with a trade-off in terms of computational time, as shown in Figure 8. The time required to solve the RO, SO, and Brute-Force algorithms is relatively close and generally lower. The DRO algorithm derives the optimal two-stage mean using fuzzy sets within the framework of the min-max theory of robust algorithms. The RaDROO algorithm considers CVaR on top of this, which further increases the complexity of the algorithm and takes more time to solve the problem.

The results obtained clearly indicate that RaDROO yields superior outcomes compared to other algorithms. It is worth noting that there is a drawback in terms of the time delay associated with obtaining the offloading decisions. However, this issue can be effectively mitigated by enhancing the performance of the managed physical devices.

Overall, although RaDROO incurs a certain time cost, it strikes a balance between computational time and risk consideration by incorporating CVaR into the decision-making process. It achieves improved robustness compared to traditional methods, while maintaining competitive performance. By considering CVaR, RaDROO effectively manages the risks associated with uncertain factors and makes informed task offloading decisions within the SAGIN environment.

## 6. Conclusions

This paper focuses on the task offloading decision problem within the SAGIN framework, considering the instability of computational resources at each node, as well as the limited channel and storage resources of ECNs. Based on this problem, we propose the RaDROO algorithm, which enhances its risk resistance by incorporating CVaR while building upon the traditional DRO algorithm. In addition, we conducted simulation experiments in a simulated environment. The algorithm demonstrated better results compared to the traditional robust algorithm, achieved comparable results to the traditional DRO algorithm, and proved the effectiveness of the algorithm.

There are several directions in which we can extend this work in the future. First, the execution time of the RaDROO algorithm is a major problem that needs to be solved; it may be solved by optimizing the algorithm architecture and using a better model-solving method. Second, the task uploads are transient, and it is possible to consider the real-time task offloading decision problem within the SAGIN framework. Third, the value of lambda is freely variable, posing a challenge in selecting a rational value. Finally, this experiment only considers the uncertainty of computational resources. In real-world environments, the storage spaces of ECNs and CSI also have uncertainties, and the impacts of these uncertainties on the offloading decision are also worth considering.

## Figures and Tables

**Table 1:** Technical methods comparison in the relevant literatures.

Literatures	Game	ML	RO	SO	DRO	Mean Risk-Aware	SAGIN
[12]	√						
[4,7]			√				
[13]		√					
[11]					√		
[6]				√			
[14,15]						√	
[5]					√		√
RaDROO					√	√	√

## Data Availability

Not applicable.
